# Microstrip Resonant Sensor for Differentiation of Components in Vapor Mixtures

**DOI:** 10.3390/s21010298

**Published:** 2021-01-05

**Authors:** Petr Slobodian, Pavel Riha, Robert Olejnik, Jiri Matyas, Rostislav Slobodian

**Affiliations:** 1 Centre of Polymer Systems, University Institute, Tomas Bata University, Tr. T. Bati 5678, 760 01 Zlin, Czech Republic; olejnik@utb.cz (R.O.); matyas@utb.cz (J.M.); slobodian.R@seznam.cz (R.S.); 2 Polymer Centre, Faculty of Technology, Tomas Bata University, nam. T.G. Masaryka 275, 760 01 Zlin, Czech Republic; 3 Institute of Hydrodynamics, The Czech Academy of Sciences, Pod Patankou 5, 166 12 Prague 6, Czech Republic

**Keywords:** microstrip resonant sensor, carbon nanotubes, polymer composites, organic vapors, vapor mixtures

## Abstract

A novel microstrip resonant vapor sensor made from a conductive multiwalled carbon nanotubes/ethylene-octene copolymer composite, of which its sensing properties were distinctively altered by vapor polarity, was developed for the detection of organic vapors. The alteration resulted from the modified composite electronic impedance due to the penetration of the vapors into the copolymer matrix, which subsequently swelled, increased the distances between the carbon nanotubes, and disrupted the conducting paths. This in turn modified the reflection coefficient frequency spectra. Since both the spectra and magnitudes of the reflection coefficients at the resonant frequencies of tested vapors were distinct, a combination of these parameters was used to identify the occurrence of a particular vapor or to differentiate components of vapor mixtures. Thus, one multivariate MWCNT/copolymer microstrip resonant sensor superseded an array of selective sensors.

## 1. Introduction

Chemical vapors alter the electrical resistance of conductive polymer composites filled with carbon nanotubes (CNTs) by interacting with their respective components. Recent research has been focused on both the underlying mechanisms and the promising applications in gas/vapor sensor fabrication as described in the reviews [1,2,3,4,5]. Prevailing efforts to enhance the sensors have been aimed at modifications of the adsorption properties of surfaces of pristine carbon nanotubes, which in turn increase the gas/vapor and CNT interactions and hence, the range of the monitored electrical resistance. The adsorption properties of CNTs depend on the availability of adsorption sites on CNTs, the impurity of CNTs (e.g., contamination with metallic particles [6]) and defects on CNTs [7]. Furthermore, the properties of pristine CNTs may be substantially altered by various modifications of their surfaces e.g., by annealing [7], acids [8], KMnO_4_ [9], fluorine [10,11], oxygen plasma [10,11,12,13], and amines [14,15,16]. Similarly, interactions between gas/vapors and polymer matrices of the respective sensors affect the resulting electrical resistance. A penetration of vapors into polymer matrices causes their swelling, which subsequently increases the resistivity of the carbon nanotube network providing an additional mechanism for detecting the presence of gas/vapors [17,18].

The properties of the numerous conductive carbon nanotube/polymer composites are used not only to build gas/vapor sensors but also sensors for strain and pressure monitoring [19,20], biological sensors [21,22], photo-sensors [23,24], etc. Conductive carbon nanotube/polymer composites also have other so-called technological properties, such as thermoelectric power generation or shape memory. Thus, embedded carbon nanotubes enabled the respective CNT/composite materials to be used as thermopiles [25], actuators [26,27], parts of field-effect transistors [28,29], etc.

Possible combinations of advantageous technological and sensing properties offer a multitude of new ways to design and manufacture advanced sensors. For example, waste heat dissipated from homoiothermic human bodies can be readily used as the source of electrical power for polymer thermoelectrics, which in turn can be used as unobtrusive low-cost self-powered sensors and integrated devices for biometric monitoring [30]. Similarly, a thermoelectric self-powered temperature sensor based on a tellurium nanowire/poly (3-hexyl thiophene) composite, in which the polymer serves as a conductive matrix and the density of the embedded tellurium nanowires determines its thermoelectric performance, is described in [31].

From the field of carbon nanotube/polymer composites, ethylene-octene copolymer (EOC) thermoelectric composites likewise offer a unique set of properties, which are not readily available in any other material. This composite can serve as a thermoelectric generator for a vapor sensor, in which the resistance varies in accordance with the presence of ambient chemical vapors and in turn changes the voltage, which has been induced by a source of heat [32]. Therefore, such a sensor does not require a power supply, but it self-produces electricity from a heat source, which can be waste heat of industrial processes, solar energy, body heat, etc.

Another advantageous combination of technological and sensing properties is employed in the microwave resonant circuit sensor coated with a carbon nanotube/conductive epoxy composite, which is used for the detection of ammonia [33]. Upon exposure to ammonia, the electrical resonant frequency of the sensor exhibits a reversible downshift. This resonant frequency downshift is attributed to changes in the effective dielectric constant of the composite owing to the adsorption of ammonia molecules onto the carbon nanotubes. A CNT-based inductor–capacitor resonant circuit with a CNT-SiO_2_ composite as a sensing layer of a passive wireless gas sensor is proposed in [34]. The absorption of different gases in the multiwalled carbon nanotubes SiO_2_ layer changes the permittivity and conductivity of the material and consequently alters the resonant frequency of the sensor. An integration of an antenna with a single-walled carbon nanotube (SWCNT) composite node for gas detection has been introduced in [35]. SWCNTs are integrated into a patch antenna design, which can be used for remote detection of ammonia gas by means of changes in its reflection coefficient corresponding to concentrations of ammonia gas [36]. Similarly, a CNT film (buckypaper) has been integrated into a tag antenna, which responds to interaction with ammonia by a shift in its resonant frequency [37]. A carbon nanotube sensor, which uses changes in its antenna reflection coefficient upon exposure to different polar and non-polar gases, is described in [38].

The major limitation of current gas/vapor sensors is the lack of selectivity [4]. The sensors tend to recognize only the occurrence of vapors, yet they may not distinguish individual compounds. We addressed this important issue in this paper and designed a microstrip resonant vapor sensor, which was able to detect not only the presence of a vapor as the resistive sensors did, but also to identify that particular vapor or differentiate components in a vapor mixture on the basis of changes in its reflection coefficient (RC) spectrum and the resonant frequency. In particular, the novel microstrip resonant sensor comprised conductive multiwalled carbon nanotubes (MWCNTs), which were embedded in the EOC matrix coated on one side of the microstrip. The microstrip adsorbed the molecules of chemical vapors, which changed its electrical resistance, impedance, and consequently, the RC spectrum. Since both the RC spectrum and the magnitude of the reflection coefficients at the resonant frequencies for each tested vapor was distinct, a combination of these parameters can be used to identify the occurrence of a particular vapor.

## 2. Material and Methods

### 2.1. Material and Sample Preparation

The thermoplastic polyolefin elastomer EOC with 45 wt% of octene content ENGAGE 8842 (Dow Chemicals, Midland, MI, USA) was used as a non-polar polymeric matrix for a particulate-filled composite. Multiwalled carbon nanotubes (Sun Nanotech Co. Ltd., Jiangxi, China) were produced by the acetylene chemical vapor deposition method and had electrical resistivity of 0.12 Ωcm and >90% purity. We determined by transmission electron microscopy individual nanotube diameters between 10 and 60 nm and lengths from 0.1 to 3 μm [39]. The maximum aspect ratio of the nanotubes was about 300.

**Figure 1:**
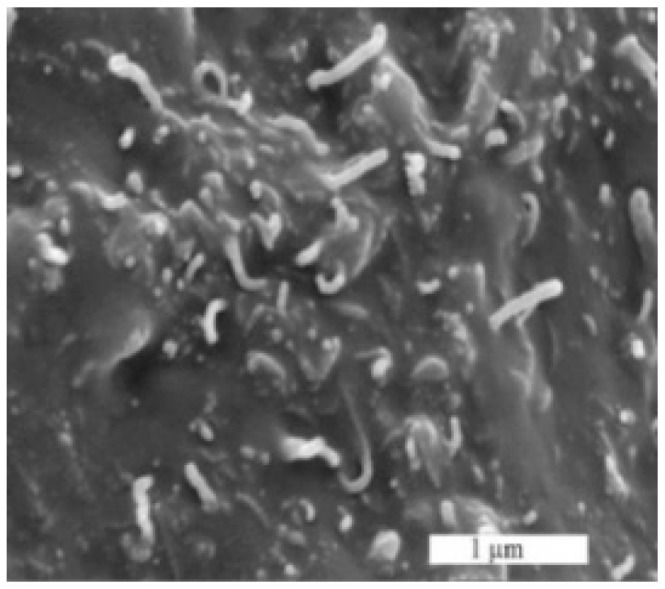
An SEM image of the MWCNT/EOC composite cross-section. The bright spots represent individual MWCNTs, which protruded from the plane of the cross-section.

To make a particulate-filled composite, EOC (20 wt%) was first dissolved in toluene of temperature 70 °C and mixed at 400 rpm for 24 h. Then, the MWCNTs were dispersed in EOC solution by a sonication using the UZ Sonopuls HD 2070 (Helago-CZ s.r.o., Hradec Kralove, Czech Republic) for 15 min at room temperature. Subsequently, the EOC solution and the appropriate MWCNT dispersion were combined and stirred at 120 rpm for two hours (shaft mixer IKA RW 1 Merck, Kenilworth, NJ, USA). The final concentration of MWCNTs in the MWCNT/EOC dispersion, which was used for the coating of the electrodes, was 36 wt%, which was well above the percolation threshold. The measured electrical conductivity of the MWCNT/EOC composites was 0.35 S/m. The cross-section structure of the MWCNT/EOC composite was analyzed by the scanning electron microscope (SEM) (NOVA NanoSEM 450, FEI Co., Lincoln, NE, USA). The corresponding micrograph is shown in Figure 1.

Interdigitated electrodes (IDEs) were prepared from Cuprextit FR-4 (a layered structure made of 1 mm thick epoxy/glass laminate coated with a 35 µm thick Cu foil) (Bungard Elektronik GmbH & Co. KG, Windeck, Germany) by etching using a 30% solution of FeCl_3_ (Sigma-Aldrich Inc., St. Louis, MO, USA) in water at room temperature. Subsequently, the rectangular electrodes (sized 25 × 20 mm) were dipped into the MWCNT/EOC dispersion and dried in an oven for 48 h at 40 °C. The ensuing MWCNT/EOC film was about 150 µm thick.

### 2.2. Electrical Resistance Measurement

**Figure 2:**
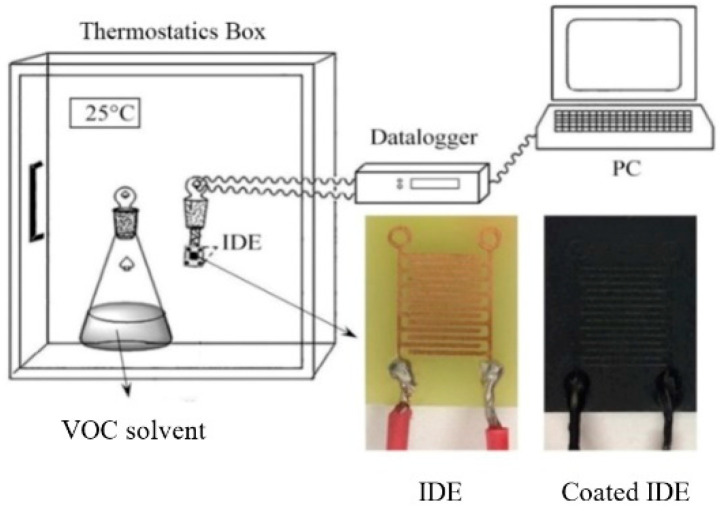
The experimental set-up for the measurement of the electrical resistance response of composite-coated on the interdigitated electrode to the ambient chemical vapors during desorption. The photos show an interdigitated electrode (IDE) and the composite-coated IDE.

The response of the composite to an adsorption and desorption of volatile organic compounds (VOCs) at 25 °C was assessed by the Multiplex datalogger 34980A (Keysight Technologies, Santa Rosa, CA, USA). In particular, the holder with the coated electrode was inserted into an airtight conical flask containing a given VOC in the liquid phase overlaid by its saturated vapor (Figure 2). After 5 min of resistance measurement, the holder was removed from the flask and the resistance during desorption was measured for the next 5 min. This procedure was repeated in five consecutive cycles. The relative humidity was 60%. The organic vapors were aliphatic hydrocarbons (heptane, pentane), aromatic hydrocarbon (toluene), ketone (acetone), and alcohols (ethanol, methanol). The polarity index of heptane is 0.1, toluene 2.4, pentane 0.0, acetone 5.1, ethanol 5.2, and methanol 5.1. The Hildebrand solubility parameter *δ* of heptane is 15.3 MPa^1/2^, pentane 14.4 MPa^1/2^, toluene 18.3 MPa^1/2^, acetone 19.9 MPa^1/2^, ethanol 26.2 MPa^1/2^, and methanol 29.7 MPa^1/2^. The saturated vapor pressure of heptane is 6.48 kPa, pentane 68.4 kPa, toluene 3.24 kPa, acetone 26.9 kPa, ethanol 6.61 kPa, and methanol 16.9 kPa at 25 °C.

### 2.3. Measurement of the Reflection Coefficient

**Figure 3:**
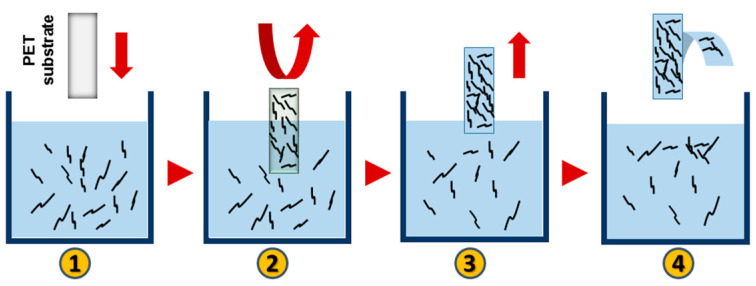
Stages of forming of the electrically conductive layer on the PET substrate by dip-coating: (1) immersion of the PET substrate in the MWCNT/EOC dispersion, (2) incubation for 10 s, (3) withdrawal (wet layer formation), (4) draining, solvent evaporation for 24 h, and peeling off of the tape with the composite layer from one side of the PET substrate.

**Figure 4:**
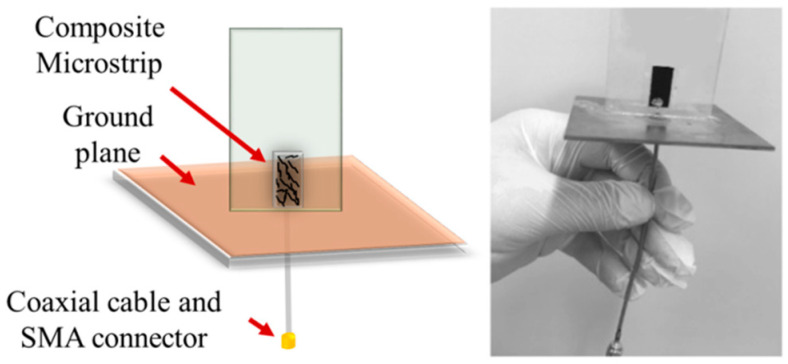
A schematic illustration and a photo of the microstrip resonant vapor sensor with the MWCNT/EOC microstrip.

The microstrip was made of the PET substrate (Fatra a.s, Napajedla, Czech Republic) coated with the MWCNT/EOC composite. The size of the 0.18 mm thick microstrip was 10 × 20 mm and its weight 0.402 g. The electrically conductive layer of the MWCNT/EOC composite was deposited on the PET substrate by a dip coating. Pristine nanotubes were dispersed in toluene and sonicated using the UZ Sonopuls HD 2070 at 50% power and 50% pulse mode at room temperature for 15 min. Then, MWCNT dispersion was mixed with the solution of EOC to prepare the coating dispersion containing 36 wt% nanotubes. Before the coating, one side of the PET foil was covered with an adhesive tape. Then, the PET substrate was immersed in the coating solution for 10 s and subsequently, left to dry at room temperature for 24 h (Figure 3). In the end, the tape was peeled off from the two-layered structure consisting of the PET substrate. The resulting unilateral composite coating of the microstrip was about 480 μm thick. The PET substrate side of the microstrip was attached to a dielectric poly (methyl methacrylate) plate (Polycasa, s.r.o., Pribram, Czech Republic) of 60 × 70 mm and thickness of 1.5 mm (Figure 4). The ground plane of the sensor was made from a Flame Retardant 4 (FR-4) epoxy substrate covered with copper (105 × 105 mm) with dielectric constant ε_r_ = 4.4. The thickness of the substrate was kept at 1.6 mm.

**Figure 5:**
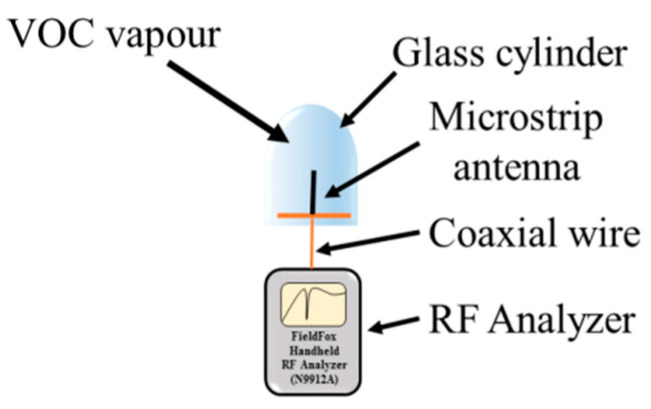
A schematic drawing of the proposed system for the measurement of the frequency dependence of the reflection coefficient by a sensor.

To assess the VOC-dependent alteration of the sensor reflection coefficient, the sensor with the composite microstrip was placed into a chamber filled with a saturated vapor of the respective VOC liquid, the layer of which was at the bottom (Figure 5). After exposure to the given vapor for the predetermined time, the power reflected from the sensor was assessed in the range from 2 MHz to 4 GHz by the N9912A FieldFox Handheld RF spectrum analyzer (Keysight Technologies, Santa Rosa, CA, USA) and quantified by the sensor reflection coefficient *S*_11_. Subsequently, the sensor was removed from the chamber and the reflection coefficient was assessed after desorption time.

## 3. Results

### 3.1. Effect of Vapors on the Composite Electrical Resistance

**Figure 6:**
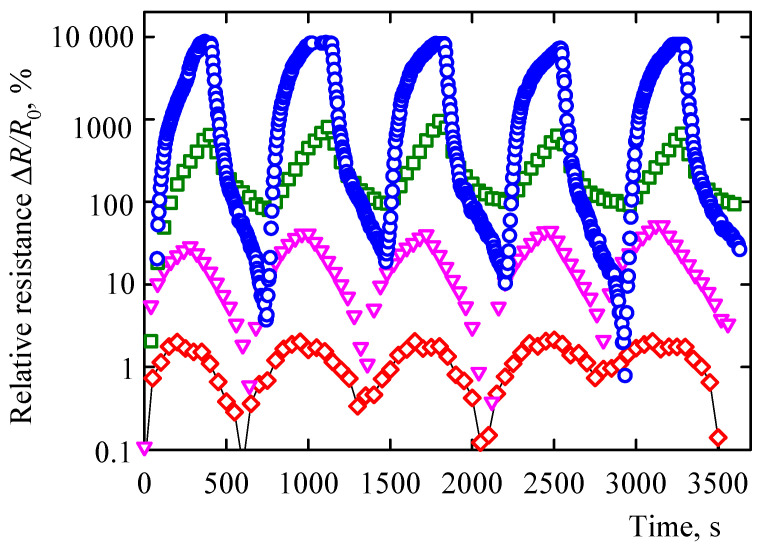
The time-dependent relative resistance of the MWCNT/EOC composite sensor in the presence of saturated vapors of heptane (blue circles), toluene (dark green squares), acetone (pink triangles), and ethanol (red diamonds) in the course of five consecutive adsorption/desorption cycles.

**Figure 7:**
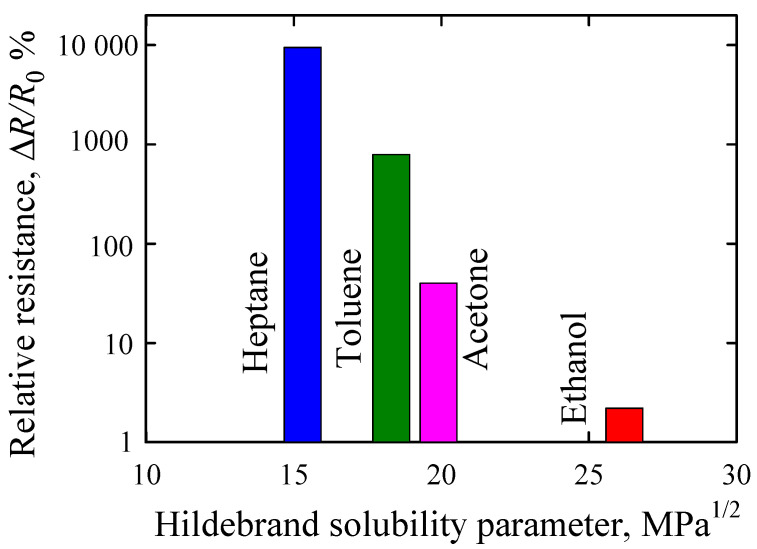
Dependence of changes in the relative resistance of the resistive MWCNT/EOC composite sensor on the Hildebrand solubility parameter of the indicated VOCs. Data are depicted as means of five cycle peak values of the relative resistances of the respective vapors.

The MWCNT/EOC composite was subjected at room temperature to organic vapors of different polarity indices. The given VOC molecules permeated into the MWCNT/EOC composite samples and increased its electrical resistance (Figure 6 and Figure 7), which was quantified by the relative electrical resistance change defined as Δ*R*/*R*_0_ = (*R − R*_0_)/*R*_0_, where *R*_0_ was the electrical resistance of the interdigitated electrode coated with the composite in the air and *R* was the resistance of the electrode during the vapor exposition. The typical kinetics of the adsorption/desorption cycles and ensuing changes in the relative resistance of the MWCNT/EOC composite sensor upon exposure to given VOC is shown in Figure 6.

The mechanism underlying the increase in resistance of the MWCNT/polymer composite in the presence of VOCs has been explained as a consequence of an adsorption of the VOCs on the MWCNT surface and of a marked expansion of the volume of the composites [1,2,3,4,12,13]. A swelling of the underlying matrix results in the formation of non-conducting layers between the embedded nanotubes, which separates the nanotubes and thus, decreases the number of conductive inter-tube contacts [17,18]. The extent of the swelling depends on the correspondence between the Hildebrand solubility parameter of the polymer and the permeating solvent [40]. For example, when the Hildebrand solubility parameters *δ* = 15.5 MPa^1/2^ for a polymer polydimethylsiloxane (PDMS) and a solvent are similar, the maximal expansion of the polymer ensues [40]. However, when the polarity and solubility of the solvent are greater than those of PDMS, the swelling of the polymer is progressively reduced [40]. By analogy, exposure of the MWCNT/EOC composite with *δ* of EOC of 16.4 MPa^1/2^ [41] to heptane with a similar *δ* of 15.3 MPa^1/2^ increased the relative resistance of the composite by almost 10,000% (Figure 7). However, penetrating vapors with higher *δ* such as toluene (*δ* = 18.3 MPa^1/2^) and acetone (*δ* = 19.9 MPa^1/2^) increased the relative resistance of the MWCNT/EOC composite to a lower extent by about 800% and 20%, respectively (Figure 7). Ethanol with *δ* = 26.2 MPa^1/2^ did not substantially alter the relative resistance of the MWCNT/EOC composite (Figure 7).

**Figure 8:**
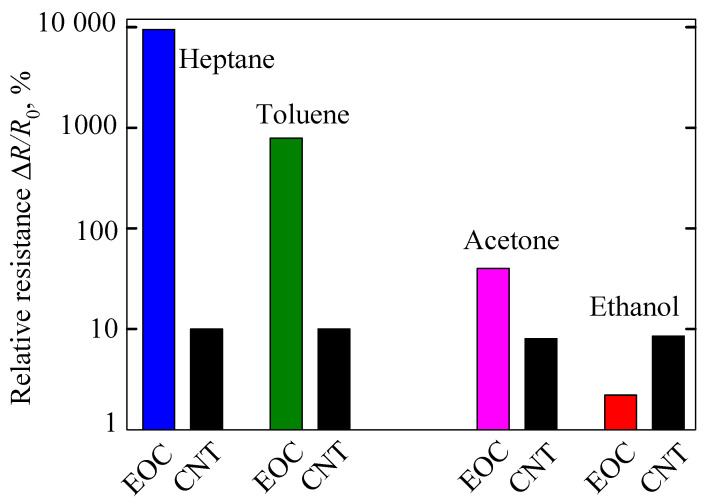
The relative resistance changes of the MWCNT/EOC composite sensor (denoted EOC) and a pristine MWCNT network (buckypaper; denoted CNT) upon exposure to indicated VOC.

Though the composite swelling played a capital role in determining its resistance increase, there was still an adsorption phenomenon affecting the composite resistance, which increased its importance as the EOC swelling was reduced in the presence of polar ethanol or acetone. Separating the influence of swelling and adsorption on the relative resistance from data on the MWCNT/EOC composite exposed to considered polar and non-polar vapors was impossible. We used an MWCNT network (buckypaper) as a close approximation of the embedded MWCNT network and measured its response upon exposure to the polar and non-polar vapors [42], and found that there were practically no differences in the effect of these vapors on the relative resistance of a freely standing MWCNT network (Figure 8). Thus, an effect of adsorption on the relative resistance change manifests indeed only when EOC matrix swelling is reduced as a consequence of the raised vapor polarity.

### 3.2. Effect of Vapors on the Microstrip Reflection Coefficient

The sensing mechanism of the resonant sensor containing the microstrip made of the MWCNT/EOC composite (Figure 4) was based on changes in reflection coefficient which indicates how much of an electromagnetic wave is reflected by microstrip impedance. If *S*_11_ = 0 dB, then all the power is reflected from the microstrip resonant sensor and none is radiated.

**Figure 9:**
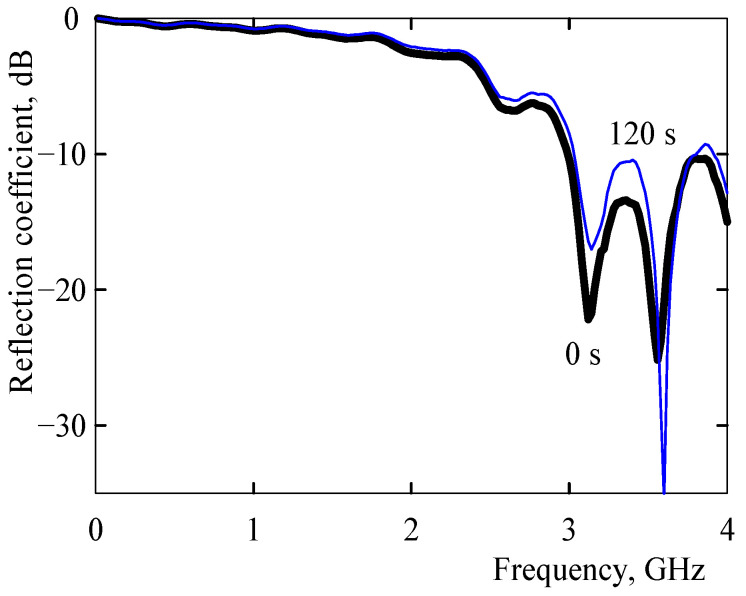
The reflection coefficient of the microstrip resonant sensor in the initial state prior to vapor exposure (thick black line) and after an exposure to non-polar heptane for 120 s at room temperature (thin blue line) within the indicated frequency range.

**Figure 10:**
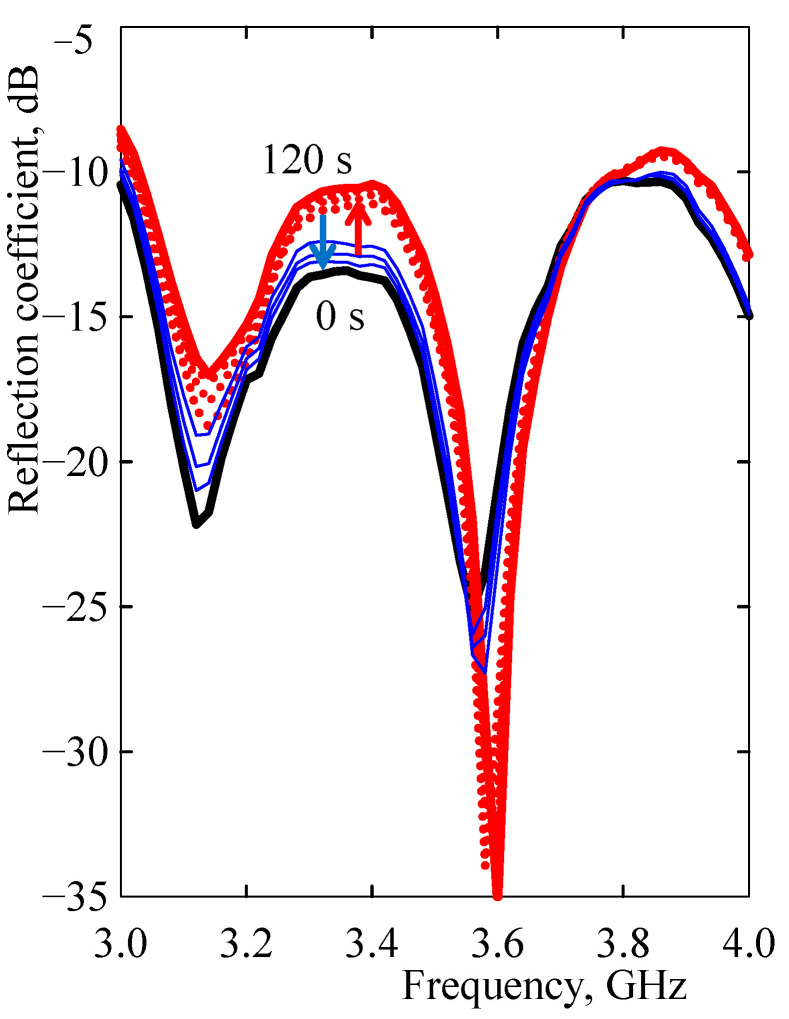
The frequency dependence of the MWCNT/EOC microstrip sensor reflection coefficient in the course of the adsorption and desorption of saturated heptane vapor. The sensor reflection coefficient spectrum in the initial state prior to vapor exposure at 0 s is represented by the thick black line. The dependence after the exposure to heptane for 15, 30, or 60 s is indicated by the dotted red lines and for 120 s by the thick red line. The dependence in the course of heptane desorption for 15, 30, 60, or 120 s is denoted by thin blue lines.

In the initial state prior to vapor exposure, there were two distinct reductions in the reflection coefficient at frequencies of about 3.1 and 3.6 GHz markedly, which were separated by a peak at about 3.35 GHz (Figure 9 and Figure 10). Upon exposure to heptane, the reflection coefficient substantially increased at about 3.1 and 3.3 GHz and markedly decreased at 3.6 GHz (Figure 9 and Figure 10). There were no differences in spectra at higher frequencies up to 18 GHz.

The dual resonant frequency mode was achieved using a microstrip with dispersed MWCNTs built into a traditional microstrip resonator, which is characterized by a single resonant frequency. Attainment of dual resonant frequency mode by incorporating the MWCNT/EOC composite was a novel promising modification of the microstrip system. The impedance of a microstrip was variable not only by the frequency of electromagnetic waves, but also by changes in its size due to exposure to vapors. For example, the average expansion of the non-polar PDMS layer (*δ* = 15.5 MPa^1/2^) by heptane (*δ* = 15.3 MPa^1/2^) is 169% [40]. The volume expansion of a similar non-polar EOC (*δ* = 16.4 MPa^1/2^) by heptane may very likely be comparable to the expansion of PDMS. The difference is that the MWCNT/EOC microstrip was attached to the PET substrate and expanded freely only in the thickness direction. The vapor-induced effect of microstrip thickness increased the resolution of the dual resonant mode. Non-polar heptane increased the value of the reflection coefficient at a frequency of electromagnetic waves of 3.1 GHz and decreased it at a frequency of 3.6 GHz (Figure 9 and Figure 10). The longer the heptane penetrated into the microstrip, the greater the difference in the values of the reflection coefficients in the resonant frequencies (Figure 10). The shift of a reflection coefficient spectrum advanced fast and after about 30 s, the spectrum was nearly the same as the final steady one after 120 s. The red arrow indicates an increase in exposure time and a shift of the spectrum of the reflection coefficient from the initial one at 0 s to the final time at 120 s. The blue arrows indicate an increase in desorption time and a shift of the spectrum of the reflection coefficient from the initial one denoted with a red thick line to the final spectrum denoted with a dark blue thick line after 120 s.

**Figure 11:**
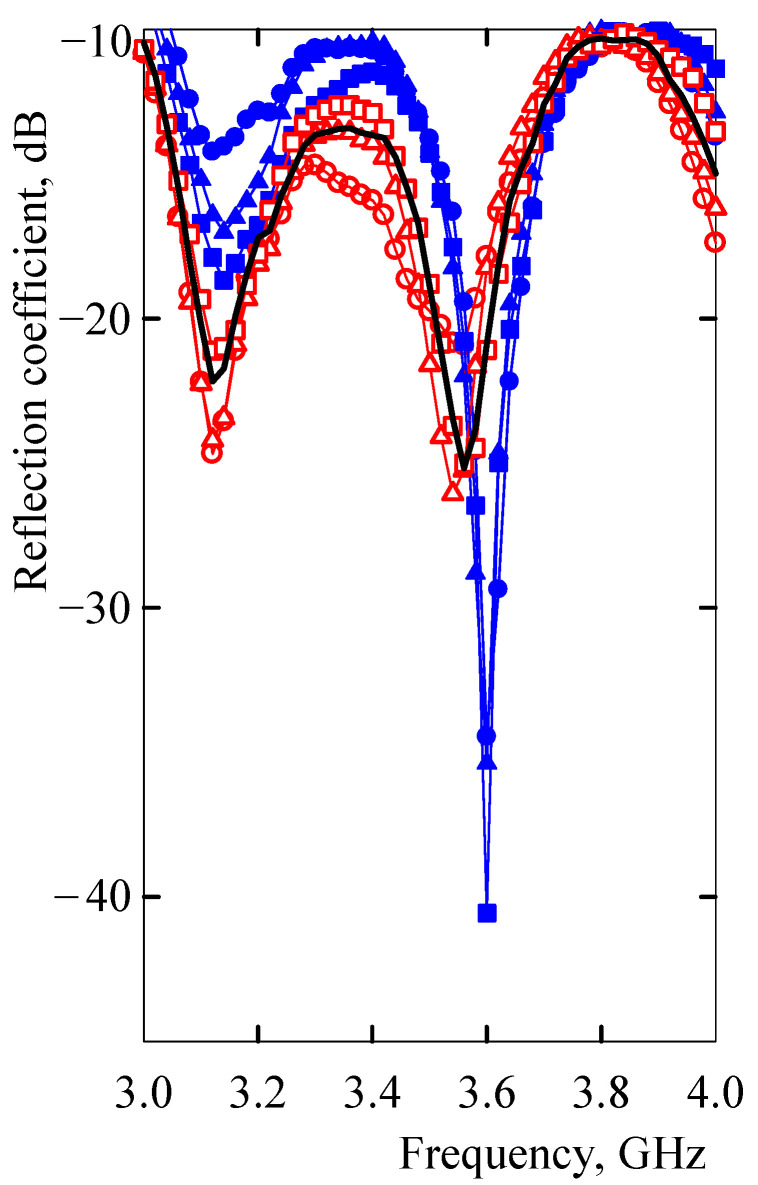
The reflection coefficient spectrum of the microstrip resonant sensor after exposure to saturated non-polar vapors (heptane, toluene, and pentane) and polar vapors (methanol, ethanol, and acetone) for 120 s. Filled circles, triangles, and squares denote heptane, toluene, and pentane, respectively. Open circles, triangles, and squares denote ethanol, acetone, and methanol, respectively. The black solid line represents the reflection coefficient spectrum unaffected by vapors.

The spectrum shape depended not only on the time of the vapor exposure but also on the corresponding solubility parameters of the respective vapors, as is shown in Figure 11. According to this figure, the relationship between the solubility parameters of vapors and a composite volumetric swelling or adsorption effects separated the RC spectra into two distinct groups. The first group included spectra affected by non-polar vapors. With *δ* values in the range 14.4–18.3 MPa^1/2^, a prevailing volumetric swelling of the microstrip composite grouped the RC spectra of these vapors. Similarly, an increased importance of molecule adsorption onto the nanotube surface when the microstrip was exposed to polar vapors grouped the RC spectra of polar vapors (Figure 11). However, the effect of polar vapor adsorption on carbon nanotube surfaces was not so significant for the change in microstrip impedance and reflection coefficient spectrum. The spectra of the reflection coefficient after exposure to polar vapors of methanol, ethanol, and acetone did not differ much from the spectrum for the resonator-based sensor unaffected by the vapors (Figure 11). The difference of the reflection coefficient from the unaffected spectrum differed at the frequency of 3.1 GHz for polar vapors by a maximum of 9% and for non-polar vapors by a minimum of 19%. At the frequency 3.6 GHz, the maximum difference for polar vapors was 16% and the minimum difference for non-polar vapors 36%. This separate response of the microstrip resonant sensor to polar and non-polar vapors was employed to differentiate components in binary and ternary mixtures in the next sections.

### 3.3. Differentiation of Components in a Binary Vapor Mixture

**Figure 12:**
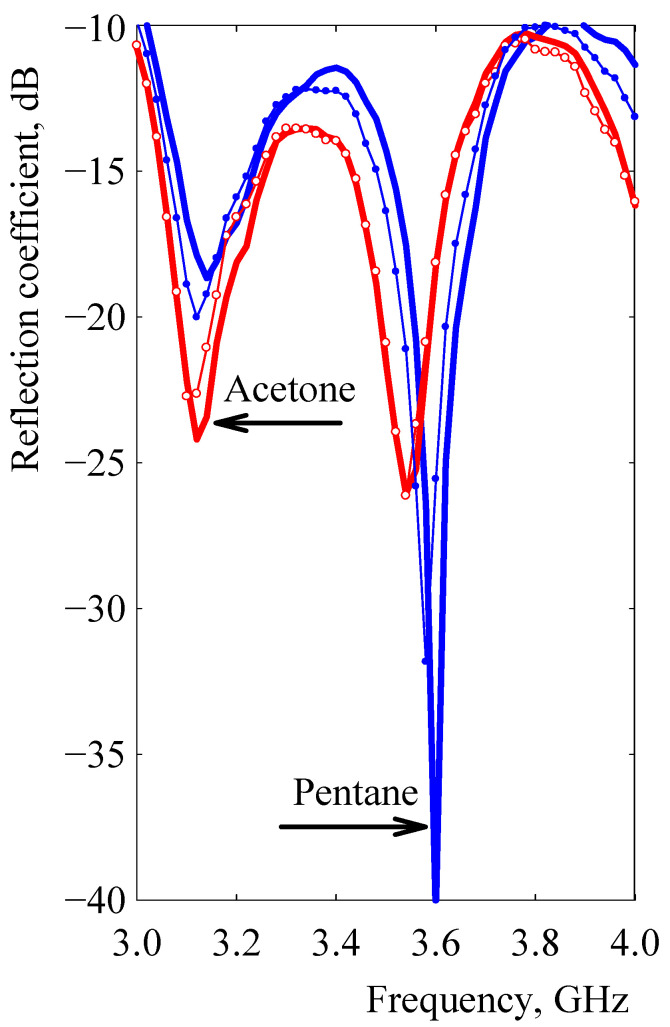
The frequency dependence of the reflection coefficient after the 120 s exposure to binary vapor mixture of polar acetone and non-polar pentane. The frequency spectrum of acetone and pentane is represented by thick red and blue lines, respectively. Filled and open circles denote acetone/pentane mixtures in the volume ratio 25:75 and 75:25, respectively.

Differences between the RC spectra of polar and non-polar vapors, presented in Figure 11 and detected by the resonant sensor with a microstrip of MWCNT/EOC composite, indicated possible use of the microstrip resonant sensor to differentiate polar and non-polar components in binary solvent mixtures. Since an array of multivariate sensors is required for differentiating mixture components [43,44], this is the first reported case of using one vapor sensor to differentiate between two vapors. A representative illustration of vapor differentiation is presented in Figure 12 for a mixture composed of pentane and acetone. The RC spectra indicate that swelling of the composite microstrip exposed to pentane decreased as the amount of acetone in the mixture was increased by 25% *v*/*v* to a pentane/acetone ratio of 75/25. The resulting mixture solubility parameter *δ*_mixture_ = ∑*x*_i_*δ*_i_ where *x*_i_ and *δ*_i_ are the component volume fraction and solubility parameter, on the other hand, increased *δ*_mixture_ = 0.25*δ*_acetone_ + 0.75*δ*_pentane_ = 15.8 MPa^1/2^ compared to *δ*_pentane_ = 14.4 MPa^1/2^. However, the reflection coefficient spectrum of the mixture was still affected mostly by 75% *v*/*v* of pentane and consequently, close to the spectrum of pentane (Figure 12). If, in the mixture at a pentane/acetone ratio of 25:75 by volume, acetone prevailed, the resulting increased solubility parameter *δ*_mixture_ = 0.25*δ*_pentane_ + 0.75*δ*_acetone_ = 18.5 MPa^1/2^ and thus, less swelling, which caused the reflection coefficient spectrum of the mixture to be affected mostly by acetone and consequently, close to the spectrum of this vapor (Figure 12). Thus, according to mixture reflection coefficient distributions, the separated contribution of swelling by non-polar pentane, and the increase in nanotube contact resistance by polar ethanol, it was possible to differentiate between these vapors and the determined prevailing ratio of one of them in the mixture.

**Figure 13:**
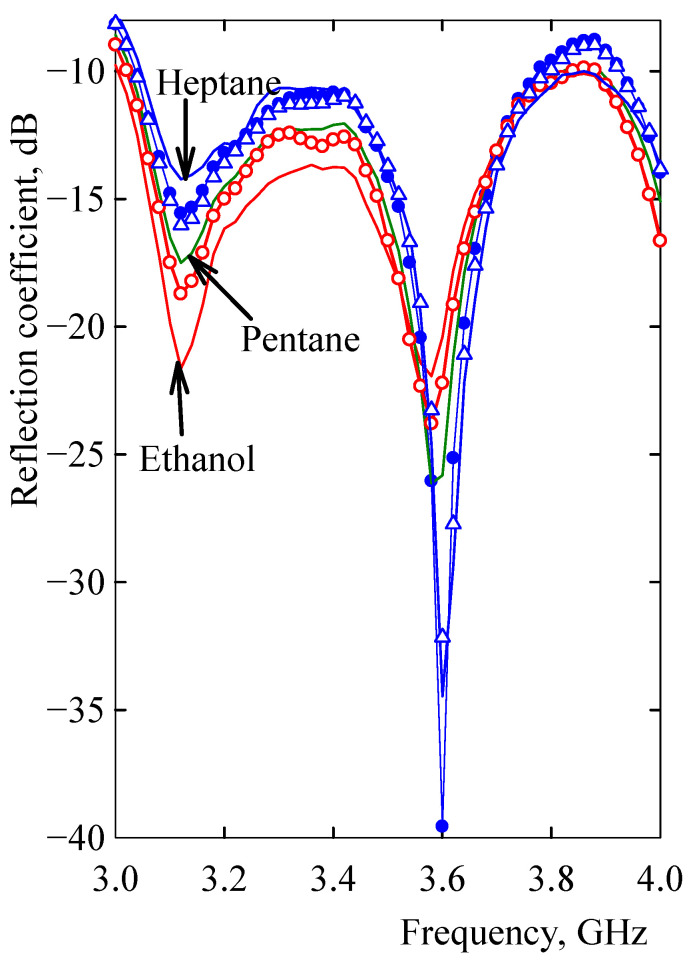
The frequency dependence of the reflection coefficient after 120 s exposure to non-polar heptane and pentane, polar ethanol, and their different mixture ratios. Thin lines are for particular pure vapors denoted in the figure. The blue line denotes heptane, dark green—pentane, and the red line denotes ethanol. Blue filled circles denote mixture 1 (heptane/pentane/ethanol in volume ratio 50:30:20), open blue triangles denote mixture 2 (30:50:20), and open red circles denote mixture 3 (10:20:70).

The composition of the pentane and acetone solvent mixture expressed in % *v*/*v* was used to identify the mixtures in Figure 12 and Figure 13 and to calculate the Hildebrand solubility parameters. However, the specific pentane/acetone volume ratio resulted in a corresponding vapor composition above the solvent mixture that penetrated the MWCNT/EOC composite microstrip. The mole fraction composition of pentane and acetone in the liquid mixed in the ratio 75:25 and 25:75 by volume was 0.657:0.343 and 0.175:0.825, respectively. The corresponding mole fractions in the vapor phase provided by Raoult’s law were 0.83:0.17 and 0.35:0.65. These mole fraction values were calculated on the assumption that the forces between molecules in the mixture are the same. However, the molecules of polar acetone interact through dipole–dipole intermolecular forces and the dominant intermolecular forces in non-polar heptane are dipole-induced dipole forces, which are not permanent, and mutual attraction of these molecules is weak. Consequently, the composition of the vapor mixture could deviate from the above-mentioned values calculated according to Raoult’s law for ideal mixtures of liquids towards a higher effect of highly volatile and MWCNT/EOC composite penetrating heptane.

### 3.4. Differentiation of Components in a Ternary Vapor Mixture

The contribution of different mechanisms to the impedance variation of the sensor microstrip made of the MWCNT/EOC composite due to polar and non-polar vapor permeation was also employed to differentiate components in a ternary mixture. For illustrating this possibility, three ternary mixtures of heptane, pentane, and ethanol were used in volume ratios 50:30:20 (mixture 1), 30:50:20 (mixture 2), and 10:20:70 (mixture 3). The obtained results shown in Figure 13 confirmed again that the extent of swelling was closely related to the value of the mixture solubility parameter, where a higher value of *δ*_mixture_ led to less matrix swelling and an increase in nanotube network resistance share on the impedance of the microstrip. Recorded spectra for the prevailing effect of non-polar heptane and pentane in mixtures 1 and 2 with *δ*_mixture_ values 15.3 and 14.4 MPa^1/2^, respectively, were located between the spectra for pure heptane and pentane and characterized by higher values of the reflection coefficient at the first resonant frequency and their lower values at the second one (Figure 13). When the volume portion of ethanol in the mixture increased over 50%, then the corresponding RC spectrum was close to one for pure ethanol and characterized by nearly the same values of the reflection coefficient at both resonant frequencies.

The specific heptane/pentane/acetone volume ratio resulted in a corresponding vapor composition above the solvent mixture that penetrated the MWCNT/EOC composite microstrip. The mole fraction composition of liquid mixture 1 was 0.37:0.27:0.36, liquid mixture 2 was 0.19:0.5:0.31, and liquid mixture 3 was 0.05:0.12:0.83. The corresponding mole fractions in the vapor phase provided by Raoult’s law were 0.09:0.66:0.25, 0.03:0.78:0.19, and 0.01:0.27:0.72 for liquid mixtures 1, 2, and 3, respectively. These mole fraction values were again calculated on the assumption that the forces between molecules in the mixture are the same. However, the molecules of polar ethanol interact through dipole–dipole intermolecular forces and the dominant intermolecular forces in non-polar heptane and pentane are dipole-induced dipole forces, which are not permanent and mutual attraction of these molecules is weak. Consequently, the composition of the vapor mixture could again deviate from the above-mentioned values calculated according to Raoult’s law for ideal mixtures of liquids towards a higher effect of especially highly volatile and MWCNT/EOC composite penetrating pentane.

## 4. Discussion

This study introduced a polymer composite consisting of an organic EOC matrix and embedded conductive MWCNTs used as a microstrip of the resonant vapor sensor for the detection of organic vapors. When the microstrip resonant sensor was exposed to polar vapors (acetone, ethanol, methanol) and non-polar vapors (heptane, pentane, toluene), the vapors permeated a composite microstrip and affected its impedance. The increase in impedance after exposition was a consequence of the polymer matrix volumetric swelling and molecule physisorption onto the nanotube surface. The extent of the swelling depended on the correspondence between the Hildebrand solubility parameter of the EOC matrix and a permeating solvent. Exposure of the MWCNT/EOC composite to non-polar heptane of *δ* = 15.3 MPa^1/2^, which had a similar solubility parameter as that of EOC of *δ* = 16.4 MPa^1/2^, increased the relative resistance of the composite by almost 10,000%. When the polarity and solubility of VOCs were greater than those of EOC, the relative resistance of the composite was less affected or unaffected by swelling, as in the case of acetone of *δ* = 19.9 MPa^1/2^ or ethanol of *δ* = 26.2 MPa^1/2^, respectively. Thus, an influence of adsorption on the relative resistance change manifested only when EOC matrix swelling was reduced as a consequence of the raised vapor polarity and the Hildebrand solubility parameter of vapors. A contribution of the prevailing effect of swelling or adsorption to nanotube surfaces to impedance variation of the microstrip was employed to identify the influence of the particular vapor or to differentiate the components of vapor mixtures.

The microstrip resonant sensor converted chemical signals elicited by the presence of chemical vapors to different spectra of the reflection coefficient. The reflection coefficient spectrum and the magnitude of the reflection coefficient at two resonant frequencies were specific for the particular vapor and related to the Hildebrand solubility parameter of this vapor. The relationship between the solubility parameters of vapors and a microstrip composite volumetric swelling or adsorption effects separated the RC spectra into two distinct groups. One group included those spectra of the reflection coefficient that were affected by a microstrip with prevailing swelling by non-polar vapors of *δ* values in the range 14.4–18.3 MPa^1/2^. The other group was formed by spectra corresponding to polar vapors with *δ* values in the range 19.9–29.7 MPa^1/2^, which diffused in a non-swollen EOC matrix and increased the microstrip impedance after vapor adsorption onto a nanotube surface. Due to two distinct underlying mechanisms of microstrip impedance variations, there were consistent differences in distributions of frequency spectra of reflection coefficients. The spectra, which responded to the prevailing effect of non-polar vapors, have shown markedly decreased magnitudes of the reflection coefficient at the second resonant frequency, while the spectra belonging to polar vapors have shown nearly the same values of the reflection coefficient at both resonant frequencies (Figure 11).

The reflection coefficient spectra provided more details about a particular vapor than a MWCNT/EOC composite resistive vapor sensor. While a response of the resistive sensor to a vapor was specified by a single value of its relative resistance change, which, for example, cannot guarantee detection of an occurrence of a particular vapor in a short response time after an alarm is triggered, the reflection coefficient spectrum was assignable to a specific permeant in a short amount of time—about 15 s (Figure 10). Moreover, due to the possibility to separate the contribution of polar and non-polar vapors into sensor impedance variation and thus, frequency distribution of the reflection coefficient, it was possible to differentiate between these vapors and to determine prevailing ratio of one of them in the mixture.

## 5. Conclusions

A new microstrip resonant sensor with an MWCNT/EOC composite microstrip for the detection of volatile organic compounds has been introduced in this paper and representative data of its ability to identify the occurrence of a particular organic vapor or to differentiate components of vapor mixtures have been shown. In particular, we assessed specific differences between sensor responses in terms of spectra of the reflection coefficient for polar and non-polar vapors. It was found that the relationship of those two types of vapors closely followed the swelling propensity of the EOC matrix. The corresponding reflection coefficient spectra made it possible to identify a particular vapor or to differentiate between those vapors in binary and ternary vapor mixtures. Usually, an array of multivariate sensors is required for differentiating mixture components. Using only one sensor instead of several ones to accomplish component identification ensures monitoring of their occurrence simultaneously without time-shifted responses of several different single-purpose sensors.

## Data Availability

The data presented in this study are available on request from the corresponding authors. The data are not publicly available due to limitations imposed by the Tomas Bata University.
